# Early Diagnosis in Ectopic Gestation

**Published:** 1912-12

**Authors:** John G. W Knoll

**Affiliations:** Buffalo, N. Y.


### Early Diagnosis in Ectopic Gestation

By JOHN G. W KNOLL, M.D.
Buffalo, N. Y.

ON December 26, 1911, and again January 10, 1912, two cases of Ectopic Gestation came under my observation and I operated them at the German Deaconess Hospital on the above dates.

Case 1. Gave a history age 28, married two months, and first menstrual period after marriage was perfectly normal and on time.

The second menstrual period was five days late and only lasted for two hours and completely stopped. Two days later the same thing occurred. Four days later, there was a sudden sharp pain in right pelvic segment followed by a profuse bloody uterine discharge; also pain in rectum and retention of urine. Pulse 110, temperature 98.5.

Physical Examination. Uterus fixed and shoved to the left side, large mass which extended from right pelvic segment high up into abdomen, considerable pain on manipulation. Intra uterine measurement 5 inches.

Operation. On opening peritoneum, the cavity was almost colorless; the entire pelvic space was covered by dense adhesions.

After breaking through these, a large amount of dark blood clots were removed, exposing the ruptured tube and its foetal contents.

She had an uninterrupted recovery.

Case 2. Gives a history married 16 years, gave birth to child 15 years ago, and has not been pregnant since.

With the exception of a profuse Leucorrhoeal discharge, pain in right pelvic segment from time to time history negative.

At last menstrual period, she flowed only four hours and this was followed by excruciating pain in right side which radiated down the right leg obliging her to remain in bed. Temperature 98.5, pulse 115.

Physical Examination. Uterus was perfectly movable. A small mass was felt in lower right pelvic segment, mostly in cul de sac. Considerable pain on manipulation. Intra uterine measurement, 5 inches.

Operation. On opening peritoneum, which was deeply stained with blood, a small mass of clots were removed from right pelvic segment and cul de sac.

The fimbriated extremity of right tube was adherant to posterior surface of uterus. The tube contained besides the foetal contents, a few blood clots.

She had an uninterrupted recovery.

The early diagnostic facts I wish to call attention to are: 1st. The normal temperature and increased pulse rate.

2nd. Sudden pain in pelvic segments following appearance of bloody uterine discharge.

3rd. Physical examinations show mass in pelvic with increased intra utrine measurement.

I might state that the other adnexia in these cases were apparently normal.

In the face of a specific G. history of husband in Case 1, in both members in Case 2, Gonococci infection was not a factor in any way to assist in the diagnosis.

